# Functional Dissection of *Streptococcus pyogenes* M5 Protein: the Hypervariable Region is Essential for Virulence

**DOI:** 10.1371/journal.pone.0007279

**Published:** 2009-10-01

**Authors:** Johan Waldemarsson, Margaretha Stålhammar-Carlemalm, Charlotta Sandin, Francis J. Castellino, Gunnar Lindahl

**Affiliations:** 1 Department of Laboratory Medicine, Division of Medical Microbiology, Lund University, Lund, Sweden; 2 W M Keck Center for Transgene Research and the Department of Chemistry and Biochemistry, University of Notre Dame, Indiana, United States of America; Columbia University, United States of America

## Abstract

The surface-localized M protein of *Streptococcus pyogenes* is a major virulence factor that inhibits phagocytosis, as determined *ex vivo*. Because little is known about the role of M protein *in vivo* we analyzed the contribution of different M protein regions to virulence, using the fibrinogen (Fg)-binding M5 protein and a mouse model of acute invasive infection. This model was suitable, because M5 is required for mouse virulence and binds mouse and human Fg equally well, as shown here. Mixed infection experiments with wild type bacteria demonstrated that mutants lacking the N-terminal hypervariable region (HVR) or the Fg-binding B-repeat region were strongly attenuated, while a mutant lacking the conserved C-repeats was only slightly attenuated. Because the HVR of M5 is not required for phagocytosis resistance, our data imply that this HVR plays a major but unknown role during acute infection. The B-repeat region is required for phagocytosis resistance and specifically binds Fg, suggesting that it promotes virulence by binding Fg. However, B-repeat mutants were attenuated even in Fg-deficient mice, implying that the B-repeats may have a second function, in addition to Fg-binding. These data demonstrate that two distinct M5 regions, including the HVR, are essential to virulence during the early stages of an infection. In particular, our data provide the first *in vivo* evidence that the HVR of an M protein plays a major role in virulence, focusing interest on the molecular role of this region.

## Introduction

A surface protein of a bacterial pathogen qualifies as a virulence factor if a mutant lacking this protein is attenuated in an animal infection model. Although many bacterial proteins fulfill this criterion, little is known about the contribution to virulence of different regions within a protein. Here, we study this problem for the M protein of *Streptococcus pyogenes* (group A streptococcus), a major human pathogen [1].

The surface-anchored M protein is a fibrillar molecule that plays a key role in host colonization and virulence [2]–[5]. The best known property of this protein is its ability to inhibit phagocytosis under nonimmune conditions, *i.e.* in the absence of opsonizing antibodies. Evidence for this property was first obtained in classical *ex vivo* studies employing whole human blood [6], [7]. More recent *ex vivo* and *in vitro* studies have ascribed additional functions to M protein, including epithelial cell adhesion [8], [9], formation of toxic soluble complexes with fibrinogen (Fg) [10], induction of T-regulatory cells [11], acquisition of surface-localized plasmin activity [12], and camouflaging of other bacterial surface components [13]. However, little is known about the function of M protein *in vivo*.

Analysis of the *in vivo* role of M protein is complicated by the fact that *S. pyogenes* is a strict human pathogen, limiting the use of animal models. Indeed, the best models available for studies of *S. pyogenes* may be two *ex vivo* models employing human material, the whole blood model [14], [15] and an organ culture model employing tonsillar tissue [16]. However, even *ex vivo* models have limitations and the use of animal models is essential [17]. Primate models for studies of *S. pyogenes* have been described, but for obvious reasons they can only be used in certain situations [5], [18], and the mouse remains the system of choice.

We used a mouse infection model to analyze the contribution to virulence of different regions within an extensively studied M protein, the M5 protein. Studies of this M protein were of general interest because many (but not all) M proteins have properties similar to M5 and bind human fibrinogen (Fg) [2], [19], [20]. Like other M proteins, M5 has an N-terminal hypervariable region (HVR), which is the target for opsonizing antibodies, and a C-terminal part that includes a relatively conserved C-repeat region [2], [19]. The region between the HVR and the C-repeats comprises the B-repeat region, which binds Fg and is required for phagocytosis resistance, as determined *ex vivo* [3], [21], [22]. We show here that both the HVR and the B-repeat region of M5 are essential to virulence during acute infection in mice.

## Results

### Experimental system: the M5 protein

**Figure 1:**
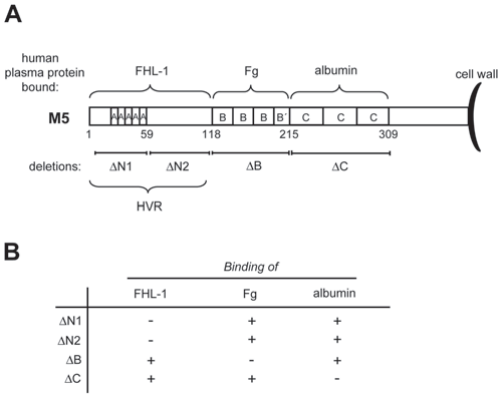
Schematic representation of the M5 protein and its ligand binding properties. (A) The M5 protein can be divided into three distinct regions: the N-terminal hypervariable region (HVR), the B-repeat region and the C-repeat region [19]. Each of these regions binds a human plasma protein, as indicated. In-frame deletions in the chromosomal *emm5* gene results in expression of the ΔN1, ΔN2, ΔB, and ΔC deletion variants. After removal of the signal peptide, M5 has a total length of 450 aa residues. Part of the C-repeat region and the C-terminal region are probably hidden in the bacterial cell wall [2]. The numbers indicate aa residues in the processed form of M5. FHL-1, factor H-like protein 1; Fg, fibrinogen. Adapted from refs. [22] and [25]. (B) Ligand binding properties of bacterial mutants used in this study. The ΔN1 and ΔN2 mutants do not bind FHL-1, the ΔB mutant does not bind Fg and the ΔC mutant is unable to bind albumin, while the regions not deleted in these mutants retain their ligand-binding properties. Based on data in ref. [25].

The M5 protein comprises three well-defined regions: the HVR, the B-repeats and the C-repeats (Figure 1A). Previous work has shown that each of these regions binds a human plasma protein. The HVR binds FHL-1, a splice variant of the complement regulator factor H (FH) [23]. The role of bound FHL-1 remains unknown [24], [25], and mice do not have FHL-1 (P. Zipfel, personal communication), implying that any role of the HVR in *S. pyogenes* M5 infected mice cannot be explained through binding of FHL-1. The B-repeat region binds Fg and is required for phagocytosis resistance, making it the only region in M5 with a well-defined function, as determined *ex vivo* [21], [22], [25]. The C-repeat region binds albumin [25]–[27], and also promotes binding to CD46, a surface-localized complement regulator present on all human cells [8], [11]. It is not known whether M5 and other M proteins bind CD46 of mouse origin, but even if this were the case, the limited tissue distribution of CD46 in normal mice [28] makes it unlikely that the interaction would be of relevance in the mouse model.

### The M5 protein binds human and mouse Fg equally well

**Figure 2:**
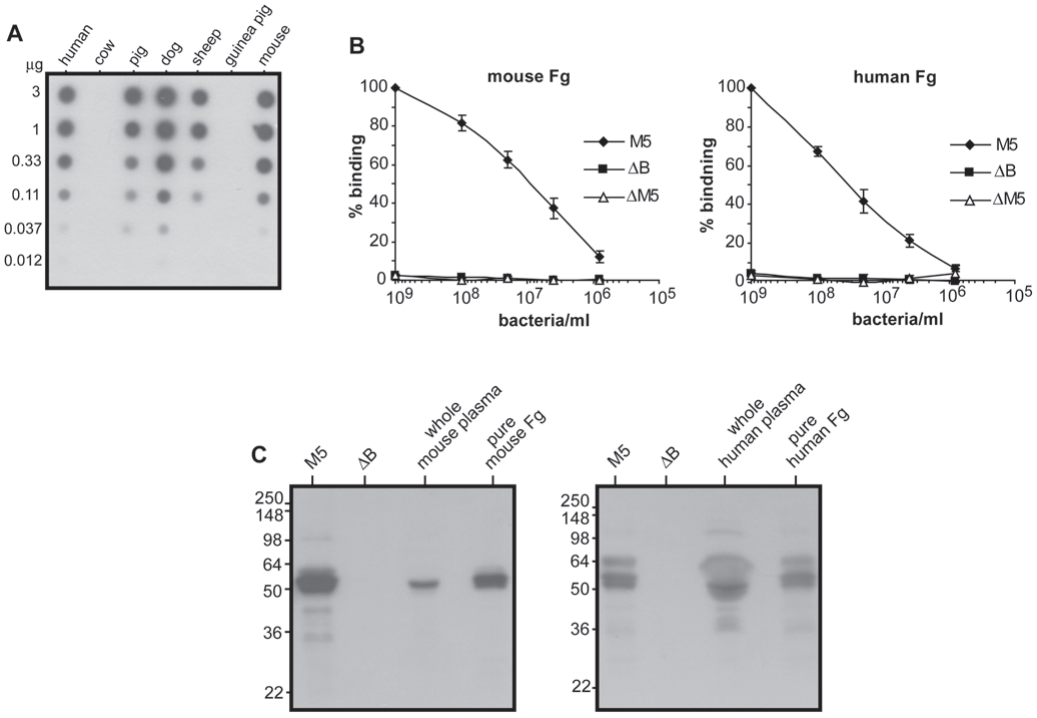
Analysis of the interaction between the M5 protein and Fg. (A) Ability of the M5 protein to bind Fgs of different origin. The immobilized Fgs were analyzed in a dot-blot, using ^125^I-labeled M5 protein as the probe. (B) Binding of ^125^I-labeled mouse and human Fg to *S. pyogenes*, as a function of bacterial concentration. M5, wild type *S. pyogenes* M5 Manfredo; ΔB, bacterial mutant lacking the B repeat region; ΔM5, mutant lacking the entire M5 protein. Maximum binding was defined as 100%. The data represent mean values with SD from three experiments with triplicate samples. (C) Ability of M5-expressing bacteria to bind Fg present in whole mouse plasma (left) or human plasma (right). Washed *S. pyogenes* bacteria from an overnight culture were incubated with plasma. Bacteria-bound proteins were eluted and analyzed by western blot, using antibodies against mouse or human Fg as the probe. Bound antibodies were detected by incubation with radiolabeled protein G, followed by autoradiography. As controls, whole plasma and pure Fg were included in each analysis, as indicated. This analysis was performed twice, with similar results.

Many M proteins bind human plasma proteins in a species specific manner and do not bind the corresponding mouse proteins [29], [30]. Because an important part of the present project was focused on Fg-binding, we analyzed whether M5 binds Fg of different origin (Figure 2A). This analysis showed that M5 binds to Fg from some but not all species analyzed and binds to both human and mouse Fg (Figure 2A). Several types of analyses subsequently demonstrated that mouse and human Fg interact with M5 in similar ways. First, radiolabeled mouse and human Fg showed binding to whole M5-expressing bacteria, but not to a bacterial mutant lacking the entire M5 protein (ΔM5) or to a mutant expressing an M5 protein lacking B repeats (ΔB) (Figure 2B). Second, western blot analysis with purified preparations of the M5 and ΔB proteins showed binding of mouse and human Fg to M5 but not to ΔB (Figure S1A). Third, mouse and human Fg were equally efficient in inhibiting the binding between human Fg and pure M5 protein, implying that the two Fgs have the same relative affinity for M5 (Figure S1B and Methods S1). Finally, M5 expressed on the bacterial surface binds mouse and human Fg present in plasma, i.e. binding occurs under physiological conditions. This was demonstrated by incubation of *S. pyogenes* in whole plasma, followed by elution of bound proteins and analysis by western blot (Figure 2C). Bacteria expressing the wild type M5 protein bound Fg in both mouse and human plasma, while bacteria expressing the ΔB protein did not.

As studies in the human system have indicated that Fg bound to M5 inhibits complement deposition [22], we analyzed whether Fg has this property also in the mouse system. When M5-expressing bacteria were incubated in mouse serum, *i.e.* in the absence of Fg, complement was deposited on the bacterial surface, but addition of pure mouse Fg at physiological concentration caused an ∼6-fold reduction in deposition (Figure S2 and Methods S1). In contrast, complement deposition on the ΔB strain was only slightly affected by the addition of Fg. Thus, binding of Fg to M5 inhibits complement deposition in the mouse system, confirming that the mouse model is suitable for studies of Fg-binding to M5.

### Mouse infections

**Figure 3:**
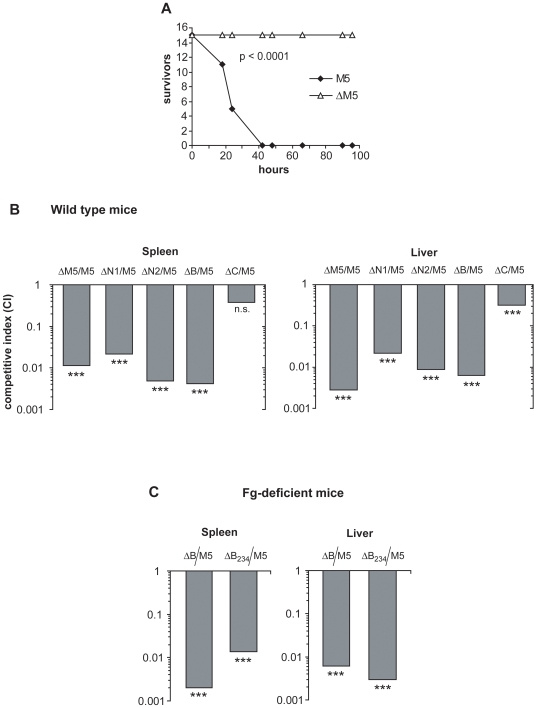
Role of M5 and different M5 regions in mouse virulence. (A) Effect of the ΔM5 mutation on lethality caused by *S. pyogenes* infection. Groups of 15 mice were infected i.p. with M5 wild type bacteria or the ΔM5 mutant. Each mouse received 3×10^7^ cfu, which corresponded to an ∼LD_90_ dose of wild type bacteria. (B) Mixed infection experiments with *S. pyogenes* mutants lacking different regions of the surface-localized M5 protein. Groups of 5 C3H/HeN mice were infected i.p. with a 1∶1 mixture of mutant and wild type bacteria, as indicated. Surviving animals (n = 3–5) were sacrificed after 44 h and spleens and livers were analyzed for presence of the two strains present in the input. Results are presented as competitive index (CI), which is defined as the ratio between mutant and wild type in the output divided by the same ratio in the input. Student's *t*-test was used for statistical analysis, in which the ratio in the input was compared with that in the output. The results were statistically significant in all cases except the spleens from mice infected with a mixture of ΔC and M5. *** = p<0.001; n.s., not significant. (C) Mixed infection experiments with Fg-deficient mice (on Balb/c background). Groups of 4 mice were infected with a 1∶1 mixture of either the ΔB mutant and wild type bacteria, or the ΔB_234_ mutant and wild type bacteria, as indicated. The deletion in the ΔB mutant covers the entire B-repeat region, while the shorter deletion in the ΔB_234_ mutant does not include the first B-repeat. The mice were sacrificed after 20 h. Analysis was performed as described in B. *** = p<0.001.

The contribution of intact M5 to bacterial virulence was first analyzed. Mice were infected with an ∼LD_90_ dose of M5-expressing wild type bacteria or a mutant (ΔM5) lacking the entire M5 protein. All mice infected with wild type bacteria succumbed to infection, whereas all mice in the group infected with the ΔM5 mutant survived (Figure 3A). These data indicate that the M5 protein is a major virulence factor in this model of lethal infection.

The contribution of different M5 regions to virulence was analyzed in a mixed infection model [31], employing a series of previously characterized chromosomal in frame deletions [22], [25] corresponding to the entire surface-exposed region of M5 (Figure 1A). The previous studies had shown that the truncated M5 proteins encoded by these mutants are expressed in normal amounts on the *S. pyogenes* surface and have the expected binding properties (Figure 1B). In particular, a deletion only abolishes ligand binding to the corresponding region but not to adjacent regions. Moreover, deletions in the HVR have little or no effect on the ability of the B-repeat region to promote phagocytosis resistance [25]. Together, these data imply that the mutants can be used to analyze the contribution of different M5 regions to mouse virulence.

The mixed infection experiments employed a ∼1∶1 mixture of mutant and wild type strain. Infected mice were sacrificed after ∼44 hours and extracts of spleens and livers were analyzed for the ratio between the two strains present in the inoculum, allowing calculation of a competitive index (CI). Of note, these experiments analyzed virulence during the early stages of an infection and under nonimmune conditions.

### Contribution of different M5 regions to mouse virulence

When mixed infection analysis was performed with the ΔM5 strain, which lacks the entire M5 protein, almost all of the recovered bacteria were wild type in both spleen and liver extracts (Figure 3B). These results indicate that the M5 protein is essential for virulence in this model and confirm the data obtained in the model of lethal infection (Figure 3A).

Analysis of the ΔN1 strain, which lacks most of the N-terminal part of the HVR, showed that also this strain was strongly attenuated, implying that the HVR is important for virulence. Similar results were obtained with a second independent HVR mutant, ΔN2, which lacks the C-terminal part of the HVR (Figure 3B). This result with ΔN2 excludes the possibility that the results obtained with ΔN1 were due to a second mutation, inadvertently introduced during mutant construction. Together, these data on ΔN1 and ΔN2 suggest that not only the N-terminal but also the C-terminal part of the M5 HVR plays a major role in virulence.

Mixed infection with the ΔB strain showed that it was as attenuated as the ΔM5 strain (Figure 3B), indicating that the B-repeat region is essential for virulence. Similar results were obtained with another shorter B-region deletion mutant in which only the C-terminal part of the B-repeat region was missing, leaving the first repeat intact, but abolishing Fg-binding (data not shown). These results in mixed infection experiments with the B region mutants were not unexpected, given the extensive data showing that this region of M5 binds Fg and is required for phagocytosis resistance *ex vivo* [21], [22], [25].

The ΔC strain, which lacks the entire C-repeat region, was only slightly attenuated in mixed infections, and results obtained for the spleen were not statistically significant. Thus, the C-repeats are of limited importance for virulence in this model (Figure 3B). Of note, the results obtained with the C-repeat mutant demonstrate that attenuation in the mixed infection model is not caused by any long deletion in the *emm5* gene.

### Role of the B-repeat region in virulence: analysis with Fg-deficient mice

The finding that the B-repeat region is essential to virulence could most simply be explained by the ability of this region to bind Fg, which has been implicated in phagocytosis resistance [22], [32]. In an attempt to obtain conclusive evidence for this hypothesis we used Fg-deficient mice, which are viable and can be used for infection studies [33], [34]. We expected that mixed infection with the ΔB mutant in Fg-deficient mice would result in similar growth for wild type bacteria and mutant, because the wild type no longer would be able to recruit Fg. Such a result would prove that the B-repeat region contributes to virulence by recruiting Fg.

If bacteria-bound Fg contributes to virulence, one might expect Fg-deficient mice to be more resistant than wild type mice to *S. pyogenes* infection, because Fg no longer could be exploited to promote virulence. However, preliminary experiments suggested that the *S. pyogenes* M5 strain studied here multiplied equally well in wild type and Fg-deficient mice, at least during the early stages of an infection. This result may reflect the homeostatic imbalance in Fg-deficient mice, causing a general increase in sensitivity to infection [33], [34]. Indeed, Fg-deficient mice showed increased sensitivity to *S. pyogenes* in a recent study employing a hypervirulent bacterial mutant [35].

Surprisingly, mixed infection experiments showed that the ΔB mutant was severely attenuated also in Fg-deficient mice (Figure 3C). Because the deletion in this mutant does not affect the known binding properties of surrounding regions in M5 ([25]; Figure 1B), it seemed unlikely that this result could be explained by an indirect effect. Nevertheless, we explored this possibility by analyzing the second B region deletion mutant described above, in which only the C-terminal part of the B-repeat region is missing. Like ΔB, this mutant (designated ΔB_234_) was attenuated in Fg-deficient mice (Figure 3C).

Because the B-repeat mutants were attenuated even in Fg-deficient mice, lack of Fg-binding is not sufficient to explain the properties of the mutants. The reason for this result remains unclear but might be explained if the B-repeats not only bind Fg but also bind a second ligand implicated in virulence, causing attenuation of mutants even in Fg-deficient mice.

### Binding properties of the B-repeat region

**Figure 4:**
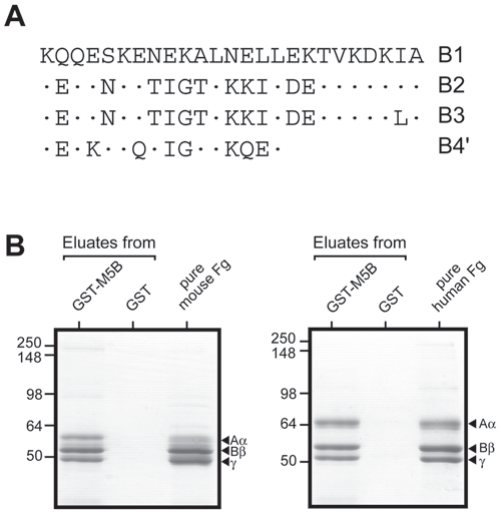
The B repeat region of M5 specifically binds Fg. (A) The aa sequence of the repeats in the Fg-binding B repeat region is shown, with identical residues indicated by a dot. The total length of the B repeat region is 91 aa [19]. (B) Affinity chromatography of mouse plasma (left) and human plasma (right) on a fusion protein derived from GST and the B repeat region of M5 (GST-M5B). The fusion protein was bound to glutathione in a column. A column containing only GST was used as control. Bound proteins were eluted and analyzed by SDS-PAGE. For comparison, pure Fg of mouse or human origin was included in the electrophoretic analysis. The ∼340 kDa Fg protein contains two Aα, two Bβ and two γ chains in both human and mouse Fg [48]. The positions of the three Fg chains are indicated. For clarity, the lower parts of the gels, containing eluted GST-M5B or GST but no other proteins, are not shown. These experiments were performed at least twice, with very similar results.

To analyze whether the B-repeat region binds a second host ligand, we analyzed the binding properties of this region in isolated form. For this purpose, we used a GST fusion protein that includes the entire 91-residue B-repeat region (Figure 4A). The binding properties of this protein, designated GST-M5B, were analyzed by passing whole plasma, of mouse or human origin, through a column containing the immobilized pure fusion protein. A column containing only GST was used as control. After washes, bound proteins were eluted and analyzed by SDS-PAGE. Eluates from the fusion protein columns only contained material that migrated like pure Fg (Figure 4B), and this material was confirmed to be Fg by western blot analysis (data not shown). These results indicate that the B-repeat region of M5 represents a distinct ligand-binding domain that only binds Fg among all plasma proteins, but are also compatible with the hypothesis that a second ligand for the B-repeats is present in tissues. However, extensive attempts to identify such a second ligand were unsuccessful (our unpublished data).

## Discussion

We used a mouse model to analyze the contribution to virulence of different regions in the *S. pyogenes* M5 protein. Studies of this M protein were of particular interest, because strains expressing M5 have been associated with rheumatic fever, which remains a major global health problem [1]. Moreover, M5 has properties similar to many other Fg-binding M proteins. At least with regard to Fg-binding, the mouse was a suitable model for *in vivo* studies of M5, because M5 binds human and mouse Fg equally well. Our results provide novel, and partly unexpected, information on the role of the HVR and the Fg-binding B-repeat region in M5.

In considering the role of the HVR in an M protein, it is important to note that the HVR cannot act only as the target for protective antibodies, but must also have an important function in the absence of protective immunity, otherwise the HVR would be eliminated by mutation. This argument does not imply that the HVR is required during acute infection, because the HVR could promote bacterial growth at later stages of an infection. For example, the HVR could act as a decoy that diverts the antibody response from other regions of the M protein or it could protect another surface component from immune attack through camouflage [13]. However, the data reported here clearly show that the HVR of M5 has an essential role during the early stages of an infection.

It has been proposed that the HVR of an M protein causes electrostatic repulsion of phagocytes, thereby promoting phagocytosis resistance [2], [36]. However, this model cannot explain the role of the HVR in M5, because the HVR of this M protein is not required for phagocytosis resistance [25]. Moreover, the requirement of the HVR in M5 for virulence cannot be explained by its ability to bind FHL-1 [23], because FHL-1 is not found in mice (P. Zipfel, personal communication). Thus, the molecular role in virulence of the HVR in M5 remains unknown.

In many M proteins the HVR specifically binds the human complement inhibitor C4BP, which contributes to phagocytosis resistance, as determined *ex vivo* [20], [37], [38]. Importantly, these C4BP-binding M proteins do not bind Fg, but C4BP bound to the HVR of an M protein may have the same function in phagocytosis resistance as Fg bound to the B-repeats of M5 [22]. Because many M proteins have HVRs that bind C4BP, we hypothesize that the HVR of M5 and similar Fg-binding M proteins also binds a host component. For example, the HVR of M5 may be required for binding to a receptor on epithelial cells. This hypothesis implies that the sequence variability in the HVRs of M5 and related M proteins may be limited by structural constraints, as must be the case for C4BP-binding HVRs [38], [39]. However, structural constraints in the HVRs that do not bind C4BP may be less severe than in those binding C4BP, as judged by analysis of diversifying selection [40] and by the demonstration of extensive subtype variation among clinical isolates [41].

A major part of this study was focused on the role of the Fg-binding B-repeats in M5. Mixed infections indicated that the B-repeat region plays a key role in virulence, as expected from its essential role in phagocytosis resistance [21], [22], [25]. This finding suggested that the B-repeats promote virulence by binding Fg, which inhibits complement deposition, but studies with Fg-deficient mice did not support this simple explanation. In contrast, our data suggest that the B-repeats not only bind Fg but also have a second and unknown role in virulence, e.g. through binding a second host ligand. This putative ligand is probably not a plasma protein, because our analysis indicated that the B-repeat region only binds Fg among all human plasma proteins. Thus, our data indicate that the B-repeat region plays a key role in virulence, while the role of Fg-binding remains unclear. This results underscores the difficulties involved in identifying the *in vivo* role of a plasma protein bound to an M protein. Indeed, the only clear evidence that a bound human plasma protein contributes to *S. pyogenes* virulence comes from studies, in a transgenic mouse model, of a plasminogen-binding M protein expressed by a minority of clinical isolates [42], [43].

**Figure 5:**
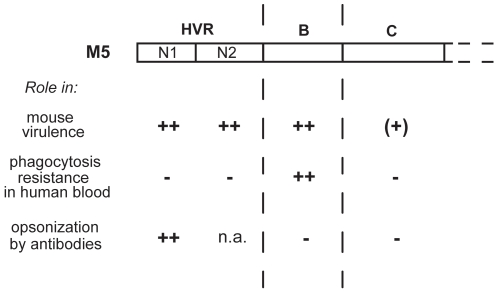
Properties of different regions in the M5 protein. This figure summarizes the role of different M5 regions in mouse virulence, phagocytosis resistance, and opsonization by antibodies. The data on mouse virulence are from this report, while the data on phagocytosis resistance and opsonization are from refs. [22] and [25]. Note that the phagocytosis and opsonization data are based on *ex vivo* assays. ++, major role; (+), very limited role; −, no role; n.a., not analyzed.

For understanding of the function of the M5 protein, it is instructive to compare the virulence data reported here with known data on phagocytosis resistance and opsonization in the M5 system (Figure 5). Mouse virulence requires the HVR and the B-repeats, as shown here. In contrast, only the B-repeat region is required for phagocytosis resistance, as determined *ex vivo* [22], [25]. Nevertheless, only antibodies to the HVR have opsonizing capacity in the *ex vivo* phagocytosis system [25]. The region in M5 that promotes phagocytosis resistance is therefore distinct from the region targeted by opsonizing antibodies. It may seem paradoxical that antibodies to the HVR are opsonizing, although the HVR is not required for phagocytosis resistance, but this finding may be explained by the surface-distal location of the HVR. *In vivo*, antibodies to the HVR may also confer protection by blocking the essential function of this region. The lack of opsonization by antibodies to the B- and C-repeat regions may be explained by the ability of bound human plasma proteins to block antibody access to these regions [25]. Together, these comparisons indicate that the three regions in M5 have distinct functions and properties.

In summary, we used a mouse infection model to analyze the contribution to virulence of three different regions in M5, a major bacterial virulence factor. Our data indicate that two of the M5 regions, the HVR and the B-repeats, contribute to virulence, while the C-repeats play a minor role, at least in the acute infection model used here. Thus, further studies aimed at identification of the *in vivo* function of the HVR and the B-repeats will be important for understanding of *S. pyogenes* pathogenesis. Such studies will also be of interest for vaccine development, because constructs derived from HVRs are being evaluated for ability to elicit protective immunity in humans [44].

## Methods

### Bacterial strains, media and plasmids

*S. pyogenes* strain M5 Manfredo, a clinical isolate recovered from a patient with rheumatic fever [19], was obtained from Dr. M.A. Kehoe. The M5-negative mutant ΔM5 has been described [23]. The isogenic mutants ΔN1, ΔN2, ΔB and ΔC [22], [25] carry in-frame chromosomal deletions corresponding to aa 11–59, 62–110, 118–210 and 215–312, respectively, in the mature M5 protein. Note that the positions of these deletions differ slightly from the regions identified in M5 (Figure 1A). A shorter chromosomal B-region mutant corresponding to aa 143–210 and covering the B2–B4' repeats, but not the B1 repeat, was constructed using previously described procedures [22]. All strains were grown in Todd-Hewitt broth supplemented with 0.2% yeast extract (THY) in 5% CO2 at 37°C without shaking.

### Proteins and antisera

The M5 protein was purified after expression in *E. coli*, using whole cell lysates and a combination of ion exchange chromatography and gel filtration, as described for other members of the M protein family [45]. The ΔB derivative of the M5 protein was purified by a similar procedure, using an *E. coli* strain carrying a pUC19 derivative encoding that protein. Protein G was from Amersham Biosciences (Uppsala, Sweden). Pure human Fg was from Enzyme Research (Swansea, U.K.). Animal Fgs were from Sigma-Aldrich (St. Louis, MO). Goat anti-mouse Fg was from Nordic Immunological Laboratories (Tilburg, The Netherlands). Rabbit anti-human Fg was from DakoCytomation (Glostrup, Denmark). Antisera specific for different regions of M5 have been described [25], except for one directed against the N2 region, which was prepared by absorbing an antiserum raised against the entire HVR with ΔN2 bacteria.

### Affinity chromatography on the fusion protein GST-M5B

For construction of the fusion protein GST-M5B, a region corresponding to aa 118–210 in the mature M5 protein was amplified by PCR from *S. pyogenes* M5 Manfredo. This M5 region corresponds to the entire 91-residue B-repeat region and two additional aa located immediately after the B-repeats. The PCR fragment was introduced into pGEX-6P-2 (Amersham, Uppsala, Sweden), yielding the expression vector pGEX-M5B, which included an introduced stop codon. The sequence of the construct was verified by DNA sequencing. The fusion protein GST-M5B was purified according to the manufacturer's instructions and dialyzed against PBS. This protein was homogeneous and of the expected size, as judged by SDS-PAGE.

For affinity chromatography, the GST-M5B fusion protein (1 mg) was immobilized in a 1 ml GSTrap column (Amersham) and 0.5 ml human or mouse plasma (diluted 5-fold in TBS) was applied. Hirudin (Refludan; Schering; 140 U/ml) was used as anti-coagulant. The column was washed with TBS (10 ml) and bound proteins (including GST-M5B) were eluted with TBS containing 20 mM glutathione. The eluate was analyzed by SDS-PAGE. As a control, plasma was applied to a column containing GST.

### Mice

Unless otherwise stated, infection experiments employed 8- to 11-week-old C3H/HeN mice, bred in the animal facility at the Medical Faculty of Lund University and maintained under pathogen-free conditions. The Fg-deficient mice used here were generated by targeted inactivation of the murine Fg γ chain [34], followed by crossbreeding of the resulting C57Bl/6 mice for 10 generations with Balb/c mice. All animal experiments were approved by the Lund/Malmö review board on animal studies and conformed to relevant regulatory standards.

### Analysis of mouse lethality

Samples of a log-phase culture of the *S. pyogenes* strain indicated were used for i.p. infection of C3H/HeN mice. Each mouse received 3×10^7^ cfu (diluted in THY), which represented an ∼LD_90_ dose for the wild type bacteria. (Note that this dose of wild type bacteria was twice as high as that used in the mixed infection experiments described below, explaining the lethality.) Deaths were recorded regularly for 96 h.

### Mixed infection experiments

Groups of 5 C3H/HeN mice were infected i.p. with a 1∶1 mixture of log-phase M5 wild type bacteria and one of the mutants ΔM5, ΔN1, ΔN2, ΔB or ΔC. This mixture was defined as the input. Each mouse received a total of ∼3×10^7^ cfu. Surviving mice (n = 3–5) were sacrificed after 44 hours and spleens and livers were collected. The organs were homogenized and samples were plated on blood agar. The bacteria in these samples, defined as the output, were analyzed for presence of the two strains present in the input. For each organ, a total of 270–450 colonies were picked and grown in short patches on THY agar plates, followed by phenotypic analysis by blotting analysis. For this purpose, a Petri dish-shaped paper (referred to as membrane below) was placed on the patches for ∼10 min, followed by incubation in a blocking solution (50 mM Tris-HCl pH 8.0 with 150 mM NaCl and 0.25% gelatin) for 1 h. After washes with TBS, the membrane was incubated for 1 h with the appropriate rabbit antiserum, diluted in TBS containing 0.25% gelatin. The antiserum was directed against an M5 region absent in the mutant strain of the input, allowing distinction between wild type and mutant bacteria. After incubation with antiserum, the membrane was washed with TBS containing 0.05% Tween-20 (TBS-T). Bound antibodies were detected by incubation of the membrane in a solution containing ^125^I-labeled protein G, followed by autoradiography. As controls, each membrane in the analysis included M5 wild type bacteria and the mutant employed in the input. All incubations were performed at RT with gentle shaking. To determine the exact ratio of wild type bacteria and mutant bacteria in the input, 270 colonies were picked and analyzed by blotting, as described above. The competitive index (CI) was defined as the ratio between mutant and wild type in the output divided by the same ratio in the input [31].

Mixed infection experiments in Fg-deficient mice were performed as described above, except that spleens and livers were harvested after 20 h.

### Statistical analysis

Competitive indices (CIs) derived from mixed infection experiments were analyzed with Student's t-test. Data from mouse lethality studies were analyzed with Fischer's exact test.

### Other methods

Dot blot and Western blot analysis were performed as described [46]. Binding assays with whole washed bacteria and radiolabeled ligands were performed essentially as described [47]. Maximal binding (defined as 100%) was ∼20% for human Fg and ∼50% for mouse Fg; such variability in maximal binding is commonly seen in work with radiolabeled proteins and reflects differences in sensitivity to the ^125^I-labeling procedure. Plasma absorption assays with whole washed bacteria were performed essentially as described [23], with hirudin as anti-coagulant.

## Supporting Information

Figure S1 Analysis of the interaction between the M5 protein and Fg. A) Western blot analysis with the purified M5 and ΔB proteins, using ^125^I-labeled mouse or human Fg as the probe. B) Inhibition of the interaction between human Fg and M5 protein. Human Fg was immobilized in microtiter wells, followed by addition of a mixture of radiolabeled M5 protein and inhibitor (mouse or human Fg). The final concentration of the inhibitor is indicated on the X-axis. Binding of M5 protein in the absence of inhibitor was defined as 100%. These data represent mean values with SD from three experiments with triplicate samples.
(3.87 MB TIF)

Figure S2 Mouse Fg bound to the B repeat region of M5 inhibits complement deposition on the bacterial surface. Samples of M5 wild-type bacteria or ΔB mutant bacteria were incubated in mouse serum, or in mouse serum supplemented with mouse Fg, as indicated. Deposition of C3 on the bacterial surface was detected with a FITC-conjugated antibody directed against mouse C3. The data presented to the left represent mean values, with SD, from three experiments. Deposition on the M5 wild-type strain incubated in serum was defined as 1.00. A representative histogram from one experiment is presented to the right. Blue line, M5 bacteria incubated in mouse serum; black line, M5 bacteria incubated in serum supplemented with Fg; green line, ΔB bacteria incubated in serum; red line, ΔB bacteria incubated in serum supplemented with Fg. IC, isotype control.
(1.81 MB TIF)

Methods S1 Contains protocols for the inhibition test in Figure S1B and the analysis of complement deposition in Figure S2.
(0.03 MB DOC)
