# Supplementary material for: Functional Dissection of Streptococcus pyogenes M5 Protein: the Hypervariable Region is Essential for Virulence
Source: PLoS One. 2009 Oct 1;4(10):e7279. doi: 10.1371/journal.pone.0007279 (PMC2749438; doi:10.1371/journal.pone.0007279)
Supplement: Methods S1 — Contains protocols for the inhibition test in Figure S1B and the analysis of complement deposition in Figure S2. (0.03 MB DOC) [file pone.0007279.s003.doc]

# Methods S1

# Inhibition tests

# The analysis was performed essentially as described [1]. Briefly, microtiter wells were coated with human Fg by incubation with 50 µl of a solution (10 µg/ml) in PBS, and blocked with PBSAT. A mixture of ^125^I-labeled M5 protein (~10,000 cpm/well) and unlabeled human or mouse Fg (final concentration 0-100 µg/ml) was added to each well. After incubation for 1 h with gentle shaking at RT and washing with PBSAT, bound M5 was detected with a γ-counter. Maximum binding in the absence of inhibitor was defined as 100%.

# Analysis of complement deposition by flow cytometry

# Analysis by FACS® analysis was performed essentially as described [2]. Washed bacteria (~5 x 10^6^ cfu in 300 µl PBS), harvested at A_620_=0.4, were mixed with a sample containing mouse serum (144 µl) and a mouse Fg solution (96 µl; 2.5 mg/ml in PBS). In controls, the mouse Fg solution was replaced with an equivalent volume of PBS. Hirudin was used as anticoagulant. The suspensions were incubated for 10 min at RT. After washing with PBS, the bacteria were resuspended in 300 µl PBS containing FITC-conjugated goat anti-mouse C3 (48 µg/ml) (ICN/Cappel, Aurora, OH). As a control antibody, FITC-conjugated goat anti-HRP [(ICN/Cappel) was used. The suspensions were incubated for 10 min in the dark at RT. Following washes with PBS, the bacteria were resuspended in PBS (800 µl) and analyzed by FACS®.

1. Morfeldt E, Berggård K, Persson J, Drakenberg T, Johnsson E, et al. (2001) Isolated hypervariable regions derived from streptococcal M proteins specifically bind human C4b-binding protein: implications for antigenic variation. J Immunol 167: 3870-3877.

2. Carlsson F, Berggård K, Stålhammar-Carlemalm M, Lindahl G (2003) Evasion of phagocytosis through cooperation between two ligand-binding regions in *Streptococcus pyogenes* M protein. J Exp Med 198: 1057-1068.
